# Genomic scars as biomarkers of homologous recombination deficiency and drug response in breast and ovarian cancers

**DOI:** 10.1186/bcr3670

**Published:** 2014-06-03

**Authors:** Johnathan A Watkins, Sheeba Irshad, Anita Grigoriadis, Andrew NJ Tutt

**Affiliations:** 1 Breakthrough Breast Cancer Research Unit, Guy’s Hospital, Kings College London, Kings Health Partners AHSC, 3rd Floor, Bermondsey Wing Guy’s Hospital, Great Maze Pond, London SE1 9RT, UK

## Abstract

Poly (ADP-ribose) polymerase (PARP) inhibitors and platinum-based chemotherapies have been found to be particularly effective in tumors that harbor deleterious germline or somatic mutations in the *BRCA1* or *BRCA2* genes, the products of which contribute to the conservative homologous recombination repair of DNA double-strand breaks. Nonetheless, several setbacks in clinical trial settings have highlighted some of the issues surrounding the investigation of PARP inhibitors, especially the identification of patients who stand to benefit from such drugs. One potential approach to finding this patient subpopulation is to examine the tumor DNA for evidence of a homologous recombination defect. However, although the genomes of many breast and ovarian cancers are replete with aberrations, the presence of numerous factors able to shape the genomic landscape means that only some of the observed DNA abnormalities are the outcome of a cancer cell’s inability to faithfully repair DNA double-strand breaks. Consequently, recently developed methods for comprehensively capturing the diverse ways in which homologous recombination deficiencies may arise beyond *BRCA1/2* mutation have used DNA microarray and sequencing data to account for potentially confounding features in the genome. Scores capturing telomeric allelic imbalance, loss of heterozygosity (LOH) and large scale transition score, as well as the total number of coding mutations are measures that summarize the total burden of certain forms of genomic abnormality. By contrast, other studies have comprehensively catalogued different types of mutational pattern and their relative contributions to a given tumor sample. Although at least one study to explore the use of the LOH scar in a prospective clinical trial of a PARP inhibitor in ovarian cancer is under way, limitations that result in a relatively low positive predictive value for these biomarkers remain. Tumors whose genome has undergone one or more events that restore high-fidelity homologous recombination are likely to be misclassified as double-strand break repair-deficient and thereby sensitive to PARP inhibitors and DNA damaging chemotherapies as a result of prior repair deficiency and its genomic scarring. Therefore, we propose that integration of a genomic scar-based biomarker with a marker of resistance in a high genomic scarring burden context may improve the performance of any companion diagnostic for PARP inhibitors.

## Introduction

Cancer is a disease of the genome. In certain types of cancers, a handful of mutations drive and accompany carcinogenesis; in others, tumor growth unfolds in the context of widespread genomic turmoil [1]. The latter scenario is the consequence of the tumor securing a mutator phenotype in which one or more of the mechanisms that preserve genomic integrity are undermined. The resultant increase in the rate of spontaneous change to the genome, a phenomenon termed ‘genomic instability’, furnishes the genetic variation that is grist to the mill of natural selection [2]. Immune responses, anti-growth signaling, and competition for space and resources all contribute to the selection of cancer cell clones with the fitness advantage to proliferate and dominate the tumor landscape [3].

Unearthing the information buried within cancer genomes will have two consequences for the management of cancer in the clinic. On the one hand, identification of the genetic abnormalities that direct the acquisition of malignant features other than the mutator phenotype may enable the selection of therapies that disrupt the relevant oncogenic pathway. On the other hand, tracing scars in a patient’s tumor genome back to particular drivers of the mutator phenotype that caused them will enable the selection of treatments that target these origins. In this review, we will focus on the latter application and, in particular, on how the genomic scars that are carved out by a deficiency in a DNA repair process known as homologous recombination (HR) may be measured and used as biomarkers or companion diagnostics for response to platinum-based chemotherapies and synthetic lethal agents such as the poly (ADP-ribose) polymerase (PARP) inhibitors.

## The need for a companion diagnostic based on homologous recombination deficiency

Familial mutations in one copy of either the *BRCA1* or *BRCA2* gene predispose patients to female breast (85% lifetime risk), ovarian (10% to 40%), male breast, pancreatic, or prostate cancer [4]. The majority of breast tumors that develop in carriers of *BRCA1* mutations - the products of which are involved in HR - are triple-negative breast cancers (TNBCs) overlapping with the gene expression-defined subtype of breast cancer known as ‘basal-like breast cancer’, whereas *BRCA2* mutation-associated breast cancers have a less restricted immunohistochemical phenotype [5-7]. As a result of the BRCA1/2-related deficiency in HR, pre-cancerous cells within at-risk organs are unable to reliably repair DNA double-strand breaks [8], resulting in genomic instability that eventually leads to cancer. These tumors are intrinsically sensitive to DNA damage response inhibitors, such as the PARP inhibitors, whose putative efficacy leverages upon a synthetic lethal effect [9] in which cell death results from mutations in two or more genes but not in each gene individually (reviewed in [10]). This phenomenon is well illustrated by PARP inhibition in BRCA1/2-deficient cells whereby PARP-dependent base excision repair and replication fork maintenance functions become critical to cell viability.

Elegant preclinical work by Bryant and colleagues [11] and Farmer and colleagues [12] demonstrating the increased sensitivity of BRCA1/2-deficient cells to PARP inhibition and the subsequent resistance to PARP inhibition on restoration of BRCA2 functionality provided the impetus for the use of PARP inhibitors in patients with BRCA1/2-associated cancers and subsequently in sporadic cancers that display ‘BRCAness’ (that is, have defective HR without germline *BRCA1/2* mutations) [13]. BRCAness can be explained by epigenetic silencing of *BRCA1*/*2* or the inactivation of several other HR-associated genes such as *PTEN*, *ATM*, *ATR*, *AURA*, *PALB2*, *BRIP*, and *RAD51* and the *FANC* family of genes [14-18]. These have been associated with several malignancies, including TNBC and sporadic high-grade serous ovarian cancer (HGSC).

Despite the early success of PARP inhibitors in demonstrating efficacy and a favorable toxicity profile in the treatment of previously heavily treated hereditary BRCA1/2-related breast and ovarian cancers [19-22], trials that expanded to include patients without *BRCA1/2* mutations were less successful. Clinical features considered surrogates for BRCAness within these trials (for example, TNBC or HGSC) might not have been sufficiently specific in predicting response to PARP inhibitors. Indeed, 50% of HGSCs are thought to be HR-deficient [23].

Recent recognition that iniparib (also known as BSI-201 or SAR240550) from BiPar/Sanofi (formerly Sanofi-Aventis, Paris, France) was erroneously considered a PARP inhibitor during its clinical evaluation within a phase III trial [24,25], and new phase I and II data reporting on the anti-tumor activity of various potent PARP inhibitors such as niraparib (MK4827) [26], BMN673 [27], and rucaparib [28] in *BRCA1/2*-mutated tumors and sporadic HGSC, non-small-cell lung cancer, prostate cancer, and pancreatic cancer, have renewed enthusiasm for PARP inhibitor drug development. Therefore, the challenge remains to develop an efficient and coordinated strategy to identify effective biomarkers such that the patients who are more likely to respond to drugs like the PARP inhibitors may be identified. The complexity of the crosstalk between DNA repair pathways suggests that assays that detect the status of multiple DNA repair pathways could prove critical for PARP inhibitor biomarker development.

## Genomic aberrations in cancer

**Figure 1:**
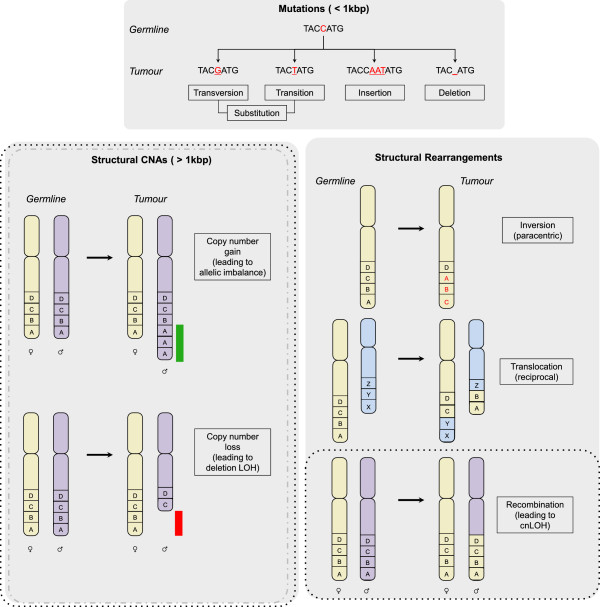
**Genomic aberrations in cancer.** Three classes of genomic aberration that develop in cancer cells are depicted: mutations of less than 1 Kbp in length (top box), structural copy number aberrations (CNAs) (bottom left box), and structural rearrangements (bottom right box). The initial state in the germline is shown followed by the corresponding change in the tumor. Mutations that affect regions of less than 1 Kbp are of three basic types: substitutions, of which there are transversions and transitions; insertions; and deletions. Insertions and deletions are often collectively termed ‘indels’. Structural CNAs are typically greater than 1 Kbp in size. One of the basic types is copy number gain. The two homologous chromosomes are shown with a gain of two further copies of region A on the paternal chromosome leading to an imbalance in the allelic ratio (1:3, maternal: paternal). The gained region is highlighted by the green bar adjacent to paternal region A. Copy number loss of regions A and B on the paternal chromosome is shown with a red bar highlighting the deleted regions. Three of the commonest types of structural rearrangement are shown, with the letters A to D and X to Z depicting defined chromosomal segments. An inversion on the same chromosome results in a change to the orientation of DNA sequences on the same chromosome either paracentrically (without crossing the centromere) or pericentrically (crossing the centromere). The inverted sequences in the tumor are shown in red. Translocations can be reciprocal or non-reciprocal and typically occur between non-homologous chromosomes (the green and blue chromosomes are non-homologous). A reciprocal translocation is shown with regions A and B exchanged for regions X and Y. Recombinations typically occur between sister chromatids where they are conservative, but can occur between homologous chromosomes (the green and purple chromosomes are homologous with green being the maternal, and purple the paternal) where recombinations at a heterozygous allelic locus can lead to cnLOH. The dotted boxes indicate where these aberrations are detectable by single-nucleotide polymorphism microarrays, whereas the grey dashed line encompasses those that can also be captured by array comparative genomic hybridization (aCGH), which does not distinguish between alleles. All forms of aberration may be interrogated by using sequencing. A, adenine; C, cytosine; cnLOH, copy number-neutral loss of heterozygosity; G, guanine; LOH, loss of heterozygosity; T, thymine.

The majority of TNBCs and HGSCs exhibit a high burden of genomic aberration. High-throughput genomic technology such as next-generation sequencing and DNA microarrays have made it possible to construct comprehensive catalogues that illustrate the complexity of such changes in those cancers. Commonly used classifications of genomic aberrations address the size and type of variation in DNA sequence (Figure 1). Mutations encompass substitutions, insertions, and deletions (collectively termed ‘indels’) that affect one or a few nucleotide bases. Depending on the location of the mutation, either the amount (mutation in a regulatory region) or the sequence (non-synonymous coding mutation) of a gene product may be affected; in either case, the impact on a protein’s function is the primary interest. Conversely, the significance of mutations irrespective of their genomic location lies with the processes by which they were generated [29,30]. Structural aberrations are operationally defined as acquired changes that exceed 1 Kbp in size. In general, two fundamental types are discernible: (a) regional copy number aberrations (CNAs), which are delineated by a gain or loss in the number of copies of a defined, subchromosomal region of DNA; and (b) structural rearrangements, which are defined by a change to the precise location or orientation of a given sequence of DNA. Of these, translocations (exchange of material between non-homologous regions of DNA), inversions (a change to the orientation of a defined sequence of DNA), and recombinations (most often used to express the exchange of material between homologous regions of DNA) are the most frequently described [31]. The potential outcome of this latter structural rearrangement is that of regional loss of heterozygosity (LOH), in which one of the parental copies of a heterozygous region of DNA is lost and the other retained. LOH that occurs as a result of a copy number loss is generally termed a ‘deletion LOH’, whereas LOH generated by an isolated recombinational event is called ‘copy number-neutral LOH’. Both copy number-neutral LOH and CNAs that lead to an imbalance in the ratio of parental alleles from the normal 1:1 constitute regions of allelic imbalance. When the rate of one or more of these structural changes increases, a cell is said to exhibit ‘structural chromosomal instability’ [32]. CNAs and LOH can also be created by alterations in the number of whole chromosomes as a result of errors in the segregation of chromosomes during mitosis. Elevation in the incidence of such events is termed ‘numerical chromosomal instability’ [32].

## Genomic scars as reporters of homologous recombination deficiency and drug response

A genomic scar can be defined as a genomic aberration with a known origin. Recent attempts at developing an assay that acknowledges the different means by which defects in HR may occur besides *BRCA1/2* dysfunction have centered around the measurement of such scars (Table 1) [29,33-35]. The major challenge in this endeavor has been to distinguish HR defect (HRD)-related genomic aberrations from the wide-ranging complexity inherent to cancer genomes. Indeed, the role played by BRCA1 in other DNA repair mechanisms such as mismatch repair and its role at stalled replication forks may obfuscate any HRD-related signal [36,37]. On the other hand, spontaneous, chance events and mutagen-induced changes have no definitive root in defective HR and yet the scars of these events may confound the quantification of a bona fide HRD. Furthermore, numerical chromosomal instability and one-off events such as whole-genome duplications and a newly described phenomenon known as ‘chromothripsis’ can all prevent the accurate measurement of HRD-related scars [32]. Chromothripsis, which is a single chromosomal shattering event followed by reconstitution of the genomic fragments, results in localized, complex rearrangements that, even if they have a basis in a targetable HR deficiency, can result in an overestimate of the gravity, and hence exploitability, of the defect [38,39]. In contrast, events that spatially overlap in such a way that only the effects of one are countable can lead to an underestimate of the extent of genomic instability [29]. In cases in which matched genomic germline data are unavailable, germline copy number variants and germline runs of homozygosity can confound CNA- and LOH-based measures of scarring, respectively.

**Table 1:** Genomic scars of homologous recombination deficiency and relationships to drug response

**Input**	**Name**	**Demonstrated objective(s)**	**Output**	**Data sets used (sample size)**	**References**
Segmented allele-specific copy number from SNP microarray data	Telomeric allelic imbalance score (*N*_tAi_)	1. Indicate sensitivity to platinum drugs	Integer between 0 and 46 per sample	Breast cancer cell lines (10 + 24)	[33,43]
		2. Indicate BRCA1/2 dysfunction		Cisplatin-1 TNBC trial (27)	
				Cisplatin-2 TNBC trial (37)	
				TCGA HGSCs (218)	
	Homologous recombination defect (HRD) score	1. Indicate HR dysfunction	Integer from 0 upper sample	MDACC ovarian cancers (152)	[32,42,43]
		2. Indicate sensitivity to platinum drugs		UPMC ovarian cancers (152)	
				TCGA ovarian cancers (435)	
				Cancer cell lines (57)	
				Cisplatin-1 TNBC trial (27)	
				Cisplatin-2 TNBC trial (37)	
				PreECOG TNBC/BRCA1/2 trial (80)	
	Large-scale transition (LST) score	1. Indicate HR dysfunction	Integer from 0 upper sample	BLBC discovery set (65)	[35,43]
		2. Indicate sensitivity to platinum drugs		BLBC validation set (55)	
				BLBC cell lines (17)	
				Cisplatin-1 TNBC trial (27)	
				Cisplatin-2 TNBC trial (37)	
	LOH clustering	1. Indicate sensitivity to platinum drugs	Three clusters of tumors: HiA,	Boston HGSCs (47)	[34]
		2. Indicate BRCA1/2 dysfunction	HiB, and Lo	Boston TNBCs (50)	
		3. Provide prognostic information		AOCS HGSCs (85)	
				TCGA HGSCs (116)	
Single-nucleotide variant calls from exome sequencing data	Total number of somatic, synonymous, and non-synonymous coding mutations (Nmut)	1. Indicate sensitivity to platinum drugs	Integer from 0 upper sample	TCGA HGSCs (316)	[44]
		2. Indicate BRCA1/2 dysfunction			
		3. Provide prognostic information			
Mutational catalogue from whole-genome sequencing data	Mutational signature 3/Mutational signature D	Indicate BRCA1/2 dysfunction	Proportion of mutational spectrum contributed by mutational signature 3 per sample	Initial breast cancer data set (21)	[1,45]
				Larger breast cancer data set (879)	

AOCS, Australian Ovarian Cancer Study; BLBC, basal-like breast cancer; HGSC, high-grade serous ovarian cancer; HR, homologous recombination; LOH, loss of heterozygosity; MDACC, MD Anderson Cancer Center; Nmut, number of coding mutation; SNP, single-nucleotide polymorphism; TCGA, The Cancer Genome Atlas; TNBC, triple-negative breast cancer; UPMC, University of Pittsburgh Medical Center.

On account of these issues, recent research has taken advantage of the allelic information and mutational context afforded by advances in single-nucleotide polymorphism (SNP) microarray and high-throughput sequencing technologies, respectively, and several measures of scarring believed to report an HRD have been developed.

### Structural chromosomal instability scars from microarrays

By training a classifier on bacterial artificial chromosome and oligonucleotide array comparative genomic hybridization (aCGH) data from *BRCA1/2* germline mutation status-annotated breast cancer data sets, several studies have demonstrated the utility of genome-wide information in identifying HR-defective tumors, which they also linked to better platinum response rates [40-42]. In general, these studies found that *BRCA1* and *BRCA2* germline-mutated cancers harbored a greater number of break points and hence copy number changes. In two studies of independent TNBC cohorts, these aCGH classifiers exhibited a sensitivity of approximately 80% in defining samples with *BRCA1* mutation [40,42]. However, in comparison with newer SNP microarray technology, aCGH presents a number of limitations, which make it more difficult to discriminate between HRD-related genomic changes and the many confounding alterations that can affect the genome, leading to poorer specificity. Specifically, the information from SNP microarray platforms makes it possible to distinguish between inherited copy number changes due to normal cell contamination and acquired DNA repair defect-related changes in cancer cells, an ability that is notably absent from aCGH analyses. Moreover, as one study described below demonstrates, the capacity to estimate tumor ploidy status from SNP microarray data - again a feature absent from aCGH data - may have implications for predicting platinum treatment outcome [35].

**Figure 2:**
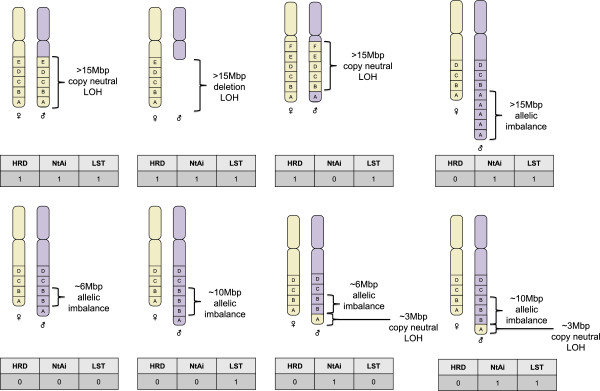
**Scoring by genomic scars of homologous recombination deficiency and drug response.** Eight examples of various forms of structural copy number aberrations and rearrangements are given, whereby each box, lettered A to F, represents a genomic segment of approximately 3 Mbp in length. Below the chromosomes, the three genomic scars - homologous recombination defect (HRD), telomeric allelic imbalance score (NtAi), and large-scale transition (LST) - are listed along with the respective integer count for the scar (0 = not seen, 1 = detected once). LOH, loss of heterozygosity.

Capitalizing on these advantages, Birkbak and colleagues [33] used SNP microarray data to test their hypothesis that the aberrant chromosomal structures formed as a result of defective HR are likely to be resolved with allelic imbalance extending from the double-strand break point to the subtelomeres of a chromosome. By scoring tumors for the frequency with which these types of genomic segment occurred, they extracted a telomeric allelic imbalance score (*N*_tAi_) (Figure 2 and Table 1) [33], which ranges from 0 to 46, with 2 being the maximum permissible contribution by each chromosome. High levels of *N*_tAi_ were shown to predict sensitivity to platinum agents in breast cancer cell lines, HGSCs and TNBCs. Moreover, tumors with mutation, promoter methylation, or low levels of mRNA for either *BRCA1* or *BRCA2* were demonstrated to have a higher burden of *N*_tAi_ than tumors without *BRCA1/2* deficiency.

In contrast, Wang and colleagues [34] discovered that clustering HGSCs according to significantly frequent regions of LOH produces three platinum response-linked groups of tumors: one harboring comparatively little LOH (Lo cluster) and two possessing high levels of LOH: the HiA and HiB clusters, distinguished by the presence and absence of 13q chromosomal loss and more frequent LOH on 5q and 17, respectively (Table 1). When the platinum response data available for three independent HGSC data sets were used, patients in the HiA cluster were found to have lower rates of resistance. In contrast, the rate of resistance was higher for the HiB and Lo clusters. Application of this LOH clustering approach to a high-grade breast cancer data set separated tumors into a Lo cluster comprising HER2- and hormone receptor-positive cancers and a Hi cluster comprising TNBCs and *BRCA1*-associated tumors. However, the relevance of the HiA-versus-HiB distinction to TNBC has yet to be investigated.

Leveraging on the known association between *BRCA1/2* deficiency and response to DNA damage-inducing drugs [21,43], Abkevich and colleagues [29], of Myriad Genetics Inc. (Salt Lake City, UT, USA), developed an HRD score defined as the number of subchromosomal segments (excluding chromosome 17) with LOH of a size exceeding 15 Mbp but shorter than the length of a complete chromosome (Figure 2 and Table 1). The objective of this score was to provide a comprehensive means of assessing defects in HR beyond sequencing of *BRCA1* and *BRCA2*. To evaluate the correlation between HRD score and HR deficiency, three independent HGSC cohorts along with 57 cancer cell lines were assessed for bi-allelic functional inactivation of *BRCA1*, *BRCA2*, or *RAD51C* through the integration of mutation, methylation, expression, and LOH data. The presence of bi-allelic inactivation of these genes was taken as a surrogate for HR deficiency. In all data sets, HRD score was elevated in HR-deficient samples, which stood in contrast to measures of whole chromosomal LOH and LOH of regions of less than 15 Mbp in length, suggesting that the maximum and minimum size thresholds employed were able to filter out aberrations because of numerical chromosomal instability and short non-HRD-related aberrations, respectively. Furthermore, in the phase II PrECOG 0105 study of gemcitabine and carboplatin plus iniparib (BSI-201) as neoadjuvant therapy for TNBC and BRCA1/2 mutation-associated breast cancer, 70% of patients with an HRD score of more than 9 responded compared with 20% of patients with an HRD score of less than 10, indicating that HRD score was significantly correlated with pathologic response. This association remained significant when patients with known *BRCA1* or *BRCA2* were excluded from the analysis [44]. Besides breast and ovarian cancers, HRD scores above 9 were characteristic for HR deficiency and were also observed in esophagus, lung, and prostate tumors as well as gastric, colon, and brain cell lines, advancing the case that HRD score has general applicability to distinct cancer types.

A separate signature of chromosomal instability, termed ‘large-scale transitions’ (LSTs), was established by using basal-like breast cancer and cell line data sets in which samples with *BRCA1* promoter methylation or *BRCA1*/*2* mutation (germline or somatic) were considered BRCA1/2-inactive [35]. For this genomic scar, copy number variant regions shorter than 3 Mb are first filtered and smoothed. This is followed by a count of the number of break points that occur between regions of at least 10 Mb in length for each chromosomal arm of a sample, with the sample’s LST score being the sum of these counts (Figure 2 and Table 1). After genomic ploidy was estimated on the basis of SNP-based microarray data, near-diploid tumors were classified as *BRCA1/2*-deficient if the number of LSTs exceeded 15. In near-tetraploid tumors, an LST cutoff value of 20 was used to segregate tumors into *BRCA1/2*-intact and *BRCA1/2*-deficient. The LST measure of HRD-related genomic scarring and its associated cutoff were found to significantly indicate *BRCA1/2* deficiency in an independent validation data set of basal-like breast cancers as well as basal-like breast cancer cell lines.

Recently, it has been shown that HRD, *N*_tAi_, and LST are highly correlated with each other and with *BRCA1/2* deficiency (*BRCA1* promoter methylation, germline, or somatic) in a breast cancer cohort that encompassed all the molecularly defined subtypes. Among TNBCs, all three scores were associated with cisplatin sensitivity [45]. Furthermore, the arithmetic mean of the three scores was even more strongly associated with *BRCA1/2* deficiency and therapeutic response.

### Sequencing-based mutational signatures

The advent of massively parallel sequencing has enabled the mutational effects of a diverse range of etiological drivers to be unraveled. By finding the total number of somatic synonymous and non-synonymous mutations (Nmut) in the exome of each ovarian tumor in a cohort of 316, Birkbak and colleagues [46] found Nmut to be higher among patients who responded well to chemotherapy (platinum agent with or without taxane) than among those who failed to respond (Table 1). Moreover, higher Nmut was observed in patients with germline or somatic *BRCA1/2* mutation. Interestingly, within the 70 ovarian tumors harboring either germline or somatic *BRCA1/2* mutation, cases that were considered chemotherapy-sensitive possessed a higher mutational burden than cases that were considered resistant, whereas in the wild-type *BRCA1/2* population, this association was not observed.

In contrast to the integer scores that Nmut and three of the SNP microarray-based scars provide, several sequence-based studies have concentrated on examining the specific type and pattern of mutations that certain genomic events leave in their wake. In the first study to use mutational context to mathematically extract signatures of mutational processes, Nik-Zainal and colleagues [47] catalogued somatically acquired mutational signatures in 21 deep-sequenced breast cancers (Table 1). These included eight TNBCs, of which five possessed germline mutation and heterozygous loss of *BRCA1*, and four non-TNBC tumors with *BRCA2* germline mutation and heterozygous loss. Interrogating the bases either side of each substitution to give a trinucleotide sequence context comprising 96 possible combinations followed by non-negative matrix factorization, the authors were able to decompose the spectrum of sequence contexts into five signatures (‘signatures A-E’) each believed to represent the scar of a distinct mutational process [1]. Hierarchical clustering of the relative contributions of these signatures to the mutational catalogue of each breast cancer revealed ‘signature A’ and ‘signature D’, representing a lesser and greater proportion of the total signature contribution, respectively, in *BRCA1*/*2*-associated tumors than in *BRCA1*/*2* wild-type tumors. Whereas ‘signature A’ exhibited enrichment for C > T conversions at XpCpG trinucleotides, ‘signature D’ displayed a relatively even distribution of mutations across the 96 trinucleotides. During investigation of the patterns of indels in the 21 tumors, two further hallmarks of *BRCA1*/2 mutation were ascertained. The first was the observation that the size of indels was typically greater in *BRCA1/2*-inactivated cancers. The second hallmark required the authors to examine whether the sequences flanking each indel were either short tandem repeats or short homologous sequences. *BRCA1/2*-inactivated tumors were differentiated from *BRCA1/2*-intact tumors by having a greater frequency of short homologous sequences adjoining indels. This observation is congruent with the notion of error-prone non-homologous end joining compensating for defective HR since such short homology-flanked indels would facilitate the joining of two non-homologous sequences through processes such as micro-homology single-strand annealing.

Following this seminal work, the repertoire of mutational signatures across 30 different cancer types was examined, and a further 16 substitution-based mutational signatures were identified (Table 1) [1]. The *BRCA1/2* defect-associated mutational signature D was relabeled ‘signature 3’ and was seen to be exclusively over-represented in breast, ovarian, and pancreatic cancers for which germline mutations to *BRCA1*/*2* have been reported to elevate the risk. Among breast tumors in the study, ‘signature 3’ was found to be operative in 255 out of 879 cases, which exceeds the estimated 5% to 10% of breast cancers accounted for by *BRCA1/2*-mutated tumors [48], supporting the case that ‘signature 3’ captures the effects of HR deficiencies attributable to a variety of means of *BRCA1/2* inactivation as well as abnormalities in the function of other genes associated with HR.

## The companion diagnostic challenge

The development of biomarkers that accurately and robustly predict treatment outcome is a key part of the drive toward personalized medicine. Already one prospective clinical trial is under way to establish HRD score for selecting appropriate patients with ovarian cancer for treatment with the PARP inhibitor, rucaparib (ClinicalTrials.gov ID: NCT01891344), and equivalent studies will be carried out as exploratory analyses in TNBCs or *BRCA1/2*-related breast cancers. Moreover, despite the sensitivity with which the genomic scars discussed predict inactivation of genes involved in HR, limitations exist to the application of these assays as a companion diagnostic for drugs that target HRDs. Unlike gene expression, which is liable to the influence of many confounding variables, genomic scars offer a comparatively stable readout of a tumor’s lifetime DNA damage repair competency, including the impact of HR inactivation where constructed to do so. Consequently, similar to other biomarkers such as estrogen receptor testing as a companion diagnostic for hormonal therapy, genomic scars are likely to prove to be high-negative predictive value (NPV) biomarkers of response to HR deficiency-targeting drugs, meaning that the great majority of patients who test negative for the biomarker will not benefit from the therapy. However, the relative stability of genomic scars is also their weakness. By chronicling the past but not documenting the present, genomic scar measures report whether or not a defect in HR has been operative at some point in tumorigenesis and not whether it remains operative at the point of treatment. A variety of mechanisms could restore HR or compensate for its loss in the aftermath of genomic scarring. Loss of *53BP1*[49] and reversion mutations to *BRCA1* and *BRCA2*[50-53] have both been demonstrated to confer resistance to platinum agents and PARP inhibitors through the restoration of HR. Pathways that operate independently of repair processes, such as drug catabolism and transporter activity, may also grant resistance [54]. To add further complexity to the issue, one study has found that upregulated activity of the c-MYC oncoprotein induces resistance to cisplatin mediated by regulation of PARP1-interacting genes [55]. Consequently, genomic scarring measures are likely to have relatively low positive predictive values (PPVs) with the consequence that a substantial number of patients who would not benefit from platinum-based agents and PARP inhibitors would be predicted to do so. Thus, although the argument for using genomic scars as a companion diagnostic may be sustainable on the basis that platinum-based agents either are the standard of care (in ovarian cancer) or have a toxicity profile at least comparable to that of standard alternatives (in breast cancer), the development of a biomarker that possesses both high NPV and PPV represents an optimal and achievable objective.

**Figure 3:**
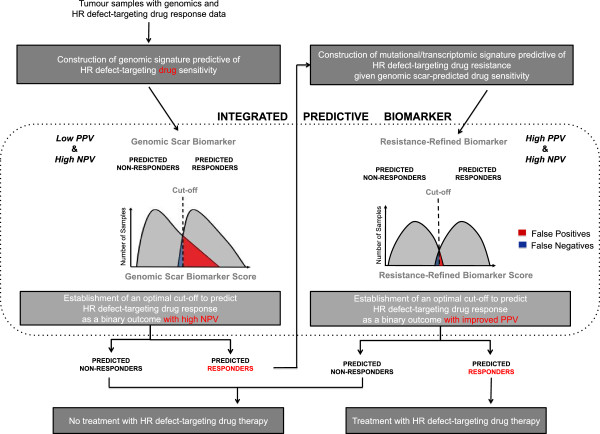
**Workflow for the development of an integrated predictive biomarker of response to homologous recombination (HR) defect directed therapy.** The workflow begins with genomics data - either sequence or single-nucleotide polymorphism microarray data - for tumor samples that have been annotated with patient response data to a given HR targeting drug therapy. After development of a genomic scar measure and a cutoff with high negative predictive value (NPV) were shown to identify non-responders but likely poor positive predictive value (PPV) due to inclusion of patients who have developed resistance (for example, 53BP1 loss) subsequent to development of the genomic scar, two groups can be identified: those predicted not to respond and those predicted to respond accepting a poor PPV. Patients in the former group should not be treated with the drug, whereas for patients in the predicted responder group, gene expression or mutation data are collected. Within the latter group, a biomarker excluding those with acquired resistance is constructed that is highly specific for response to the drug, better dichotomizing patients into those who do and those who do not benefit. By combining the genomic scar biomarker with the resistance-refined biomarker, the resultant two-step companion diagnostic should possess both high NPV and high PPV.

To address this, the development of a genomic scar-based predictive biomarker could be followed by the construction of a second biomarker by using only the population for which the genomic scar predicts drug efficacy (Figure 3). By looking within a genomic scar-predicted responder population, the signal from resistance mechanisms that specifically operate within a HR-deficient setting should be stronger than if the population was taken as a whole. Mutational data could reveal reversions in a suite of HR-related genes, whereas transcriptional data might uncover the elevated expression of genes that compensate for HR impairment. Coupling the high-NPV genomic scar biomarker with a high-PPV post-genomic scar biomarker into an integrated biomarker would thus capture the best of both approaches (Figure 3).

## Conclusions

Although targeting DNA repair deficiencies in cancer has been a mainstay of the therapeutic oncology armamentarium for decades, this has been more through serendipity and observation of average effects in populations than by mechanistic DNA repair activity-informed design. Consequently, the approach has lacked a personalized medicine companion diagnostic strategy. Consistent with the requirement of the US Food and Drug Administration for every new drug to be accompanied to market by a biomarker that predicts its effectiveness, the rapidity with which PARP inhibitors and now genomic scars have been brought from concept to clinical trial reflects the current interest in selecting patients for whom administration of a drug that impacts the DNA damage response is predicted to be clinically beneficial. However, therapies directed at HRDs are not the only examples of therapy that could be individualized by using genomic scar-based biomarkers. Any flaw in the genomic maintenance machinery that (a) can be capitalized on therapeutically and (b) leaves an imprint in the genome that is detectable through current techniques and technologies is ripe for the development of a genomic scar to predict drug response. In compiling a list of 21 validated mutational signatures, researchers have already taken the first steps toward the goal of constructing a repertoire of integrated predictive biomarkers [1]. One example outside the context of HR deficiency is that of Alexandrov and colleagues’ ‘Signature 6’ [1], which was found to be associated with a defect in DNA mismatch repair. Such a signature may in turn predict the effectiveness of drugs like methotrexate, which has been shown to be selectively effective in mismatch repair-deficient cancer cells [56]. The next steps therefore will require the characterization of the etiologies behind every one of these signatures and, in the case of SNP microarray-based scars, the expansion of our understanding of the interaction between the scar repertoire and the presence of other targetable deficiencies in the DNA maintenance machinery.

## Note

This article is part of a series on *‘Recent advances in breast cancer treatment*’*,* edited by Jenny Chang. Other articles in this series can be found at http://breast-cancer-research.com/series/treatment.

## Abbreviations

aCGH: array comparative genomic hybridization; CNA: Copy number aberration; HGSC: High-grade serous ovarian cancer; HR: Homologous recombination; HRD: Homologous recombination defect; LOH: Loss of heterozygosity; LST: Large-scale transition; Nmut: Number of coding mutation; NPV: Negative predictive value; NtAi: telomeric allelic imbalance score; PARP: Poly (ADP-ribose) polymerase; PPV: Positive predictive value; SNP: Single-nucleotide polymorphism; TNBC: Triple-negative breast cancer.

## Competing interests

The authors declare that they have no competing interests.
